# Height Velocity in Pediatric Cystic Fibrosis Under Triple CFTR Modulator Therapy: A Real-Life Monocentric Experience

**DOI:** 10.3390/jcm14155259

**Published:** 2025-07-25

**Authors:** Alessandra Boni, Francesco d’Aniello, Grazia Ubertini, Marco Cappa, Fabiana Ciciriello, Fabio Majo, Luca Cristiani, Federico Alghisi, Enza Montemitro, Sergio Bella, Matteo De Marchis, Renato Cutrera, Alessandro G. Fiocchi

**Affiliations:** 1 Pulmonology and Cystic Fibrosis Unit, Bambino Gesù Children’s Hospital, IRCCS, 00165 Rome, Italy; fabiana.ciciriello@opbg.net (F.C.); fabio.majo@opbg.net (F.M.); luca.cristiani@opbg.net (L.C.); federico.alghisi@opbg.net (F.A.); enza.montemitro@opbg.net (E.M.); sergio.bella@opbg.net (S.B.); matteo.demarchis@opbg.net (M.D.M.); renato.cutrera@opbg.net (R.C.); 2 Endocrinology and Diabetology Unit, Bambino Gesù Children’s Hospital, IRCCS, 00165 Rome, Italy; graziamaria.ubertini@opbg.net (G.U.); marco.cappa@opbg.net (M.C.); 3 Allergy Unit, Bambino Gesù Children’s Hospital, IRCCS, 00165 Rome, Italy; alessandro.fiocchi@allegriallergia.net

**Keywords:** growth, ETI, pediatric population

## Abstract

**Background/Objectives:** Cystic fibrosis (CF) is a multi-system disorder characterized by chronic respiratory failure, malnutrition, and impaired growth. Achieving linear growth above the 50th percentile is associated with better pulmonary outcomes. Since October 2022, Elexacaftor/Tezacaftor/Ivacaftor (ETI) has been approved in Italy for children aged ≥6 years. However, data on its impact on height velocity (HV) remain lacking. This study aims to evaluate growth patterns by HV and explore differences according to the CFTR variant genotype. **Methods:** We conducted a prospective single-center study at the CF Unit of Bambino Gesù Children’s Hospital involving 24 children aged 6–11 years eligible for ETI treatment. Baseline assessments included height, weight, body mass index (BMI), bone mineral density (BMD), body composition (via bioelectrical impedance analysis, BIA), and muscle strength (one-minute sit-to-stand test (1STST)). Height, weight, HV, and BMI standard deviation scores (SDS) were calculated for the 6 months before and after ETI initiation. **Results:** The mean age of the cohort was 8.7 ± 1.9 years (F/M: 12/12), with most patients naïve to CFTR modulators. A significant increase in HV was observed post-ETI: from 4.2 ± 2.0 cm/year (−1.96 ± 2.4 SDS) in the 6 months before treatment to 7.1 ± 3.0 cm/year (+1.5 ± 3.7 SDS) after treatment initiation (*p* < 0.0001). Patients with F508del/minimal function (F/MF) genotypes (n = 11) showed significantly greater HV compared to those with F508del/F508del (F/F, n = 5) and F508del/residual function (F/RF, n = 8) genotypes (*p* < 0.0001). No significant differences were observed among genetic groups in baseline BMD or lean mass. **Conclusions:** ETI treatment significantly and rapidly improves HV in children with CF, particularly in those with F/MF genotypes. These findings underscore the role of CFTR modulator therapy in promoting linear growth, a key indicator of health in pediatric CF populations.

## 1. Introduction

Cystic fibrosis (CF) is a rare, life-limiting autosomal recessive genetic disorder caused by mutations in the gene encoding the chloride-conducting transmembrane channel, known as CFTR, that regulates anion transport across multiple epithelia [1].

Malnutrition and poor growth have been key features of CF due to maldigestion and malabsorption [2]. CF children typically have reduced neonatal length and remain in the lowest growth percentiles until puberty, when severe growth restriction, delayed puberty, and reduced final stature become evident [3]. These findings suggest that CF affects height gain starting prenatally, through a mechanism that is not yet fully understood.

Although the nutritional landscape in CF has evolved progressively over the decades with the introduction of pancreatic enzyme replacement therapy, the implementation of neonatal screening programs, and aggressive guideline-directed nutritional interventions, childhood growth is still impaired in many people with CF (pwCF). In fact, despite having genetic potential and growth rates comparable to average healthy peers, their final height is usually lower than that of the general population [4].

Thus, achieving normal linear growth is an important therapeutic goal in children with CF, as maintaining height above the 50th percentile is also associated with a better lung function [5,6]. As demonstrated, children who have maintained growth parameters above the 50th percentile have a higher expected FEV1 at 6–7 years of age compared with children whose growth has declined by 10% or remained relatively constant during the first 6 years of life [6]. In the era of new CFTR modulators, the nutritional status of pwCF has also improved.

The use of the triple combination Elexacaftor/Tezacaftor/Ivacaftor (ETI) is thought to have a major impact on CFTR channel function, but its effect on height has yet to be determined [5].

In Italy, ETI was approved in October 2022 for children aged 6–11 years with at least one F508del mutation [7]. The growth outcomes of ETI reported in the registered trials focused only on BMI, weight, and height-for-age z-score [8]. There are no data on the impact of ETI on height velocity (HV), which is an acceptable tool for the healthy state of growing children. No data are reported either in the trial or in real-life to date [8].

The main objective of our study is to analyze the real impact of initiating ETI on nutritional and auxological parameters in a pediatric population in the first six months of treatment. A secondary aim was to identify differences in the increase in HV between different subgroups of the population based on genetic characteristics.

## 2. Materials and Methods

A single-center prospective study was conducted at the CF Unit of Bambino Gesù Children’s Hospital from October 2022 to May 2023. We included all CF patients eligible for ETI, starting from 6 years of age. Both CFTR modulator-naïve patients and those on previous therapy with other modulators were included. Patients were recruited during routine outpatient visits for the follow-up of the underlying chronic disease. Inclusion criteria included age between 6 and 11 years and having at least one F508del mutation, according to national guidelines for ETI therapy eligibility. Patients with a history of organ transplantation and severe hepatic impairment (Child-Pugh class C) were excluded. All eligible patients meeting these criteria were prescribed the therapy, as no patients met the exclusion criteria or declined treatment. Data were collected retrospectively from medical records. All patients (or their guardians) provided informed consent, and there were no drop-outs.

Anthropometric data, including height, weight, BMI, and HV were collected and assessed for all patients at the start of treatment (defined as T0) and before and after 6 months of clinical and auxological evaluation (defined as T (−6 months) and T (+6 months), respectively) as the standard of pediatric care. Height measurements were obtained by the mean of three consecutive measurements taken by the same physician using a scale and stadiometer. To avoid confounding, height, weight, and BMI measurements were standardized using standard deviation scores (SDS) derived from the Italian growth chart developed by Cacciari et al. [9]. Additionally, HV SDS were obtained from the study by Tanner et al. [10]. A distinction in pubertal stage was created based on Tanner staging [11]. Patients were classified as pre-pubertal if they had a Tanner staging of B1 or G1 for females and males, respectively. Patients were classified as pubertal if they had a Tanner staging above one for both genders.

Bone mineral density (BMD) scanning and body composition assessment were conducted at T0 using a Dual-energy X-ray Absorptiometry (DXA) machine (Hologic; Horizon^TM^ QDR Series, model Horizon W (S/N300245M), Marlborough, MA, USA) following standard protocols. Participants were placed in a supine position on the scanning table, ensuring proper alignment with the X-ray beam. Lumbar and total body scans were performed in accordance with manufacturer specifications. Data acquired from the DXA scans, including BMD values and z-scores (lumbar and Total Body Less Head (TBLH)), lean mass, fat mass, and other parameters, were recorded for subsequent analysis.

Additionally, Bioelectrical Impedance Analysis (BIA) was utilized to evaluate body composition at T0, employing a simultaneous direct segmental multi-frequency analysis method (LookIn’Body 120). Participants were instructed to stand on the device’s footplates for analysis. Measurements of lean mass, fat mass, bone mass, and total body water were obtained as per the manufacturer’s guidelines.

Before both DXA scans and BIA measurements, participants were advised to abstain from food and drink for a minimum of 4 h and to remove any metallic objects or jewelry.

Following the literature, we analyzed the muscle weakness using the one-minute sit-to-stand test (1STST) at the baseline evaluation [12]. As reported by Strassman et al., 1STST was performed using a 46 cm high chair with no armrests to stand fully up (legs straight) and then sit down (ensuring that the buttocks touched the chair and the knees were at a 90° bend) as many times as possible within one minute [13].

Data were subjected to statistical analysis with Shapiro−Wilk to test the sample distribution. The demographic and clinical characteristics of patients were reported as counts and proportions for categorical data, and as mean ± standard deviation (SD) for normally distributed continuous data or median with interquartile range (IQR) for non-normally distributed continuous data. The paired Student’s *t*-test was used to compare normally distributed continuous data, and the Wilcoxon matched-pairs signed rank test was used to compare non-normally distributed data. When we needed to compare variables across multiple time points, we utilized repeated measures one-way ANOVA. A simple linear regression was used to analyze the linear relationship between the dependent variables and independent variables.

At a significance level of 0.05, the Bonferroni-corrected *p* value was determined based on the number of tests performed. Statistical processing of the data was performed using GraphPad Prism (version 10.2.1).

The parents of all patients provided written informed consent to participate in the study and were informed about the possibility of waiver of consent at any time.

The study adhered to the revised Declaration of Helsinki and was approved by the local Ethical Committee of Bambino Gesù Children’s Hospital (2304_OPBG_2020). The structure of this article is based on the Strengthening the Reporting of Observational studies in Epidemiology (STROBE) checklist.

## 3. Results

A total of 24 pwCF were identified as eligible for the study. The mean age was 8.7 years (6.44–11.82 years with a SDS 1.9 years) at T0 with a balanced gender distribution of 12 males and 12 females. Most of the population has a mutation with minimal function in combination with F508del (45.8%), and pancreatic insufficiency (91.7%). Pseudomonas aeruginosa colonized only 16.7% of the sample; 8.3% of patients had non-tuberculous Mycobacterium colonization. As expected by age, the entire population had a good lung function with a low average annual exacerbation rate of 0.3 per year.

Regarding the auxological parameters at T0, the median BMI was 16.6 (SDS 1.8) Kg/m^2^ and the median height was SDS 10.6 cm. The auxological data across different time points are reported in Table 1.

Only three patients were defined as pubertal according to the Tanner staging.

The majority of the population was naïve to other modulator CFTRs (79.2%). The remaining patients were 4/5 previously on Lumacaftor/Ivacaftor therapy and 1/5 on Ivacaftor.

The demographic characteristics of the sample are presented in Table 2.

**Figure 1:**
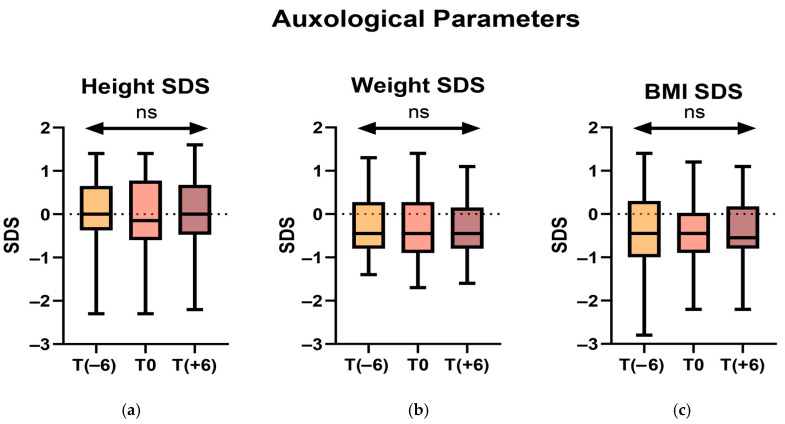
Comparison of height SDS (**a**), weight SDS (**b**), and BMI SDS (**c**) across all time points. (ns = not significant).

No statistically significant difference was found between any of these parameters across all observations (Figure 1).

**Figure 2:**
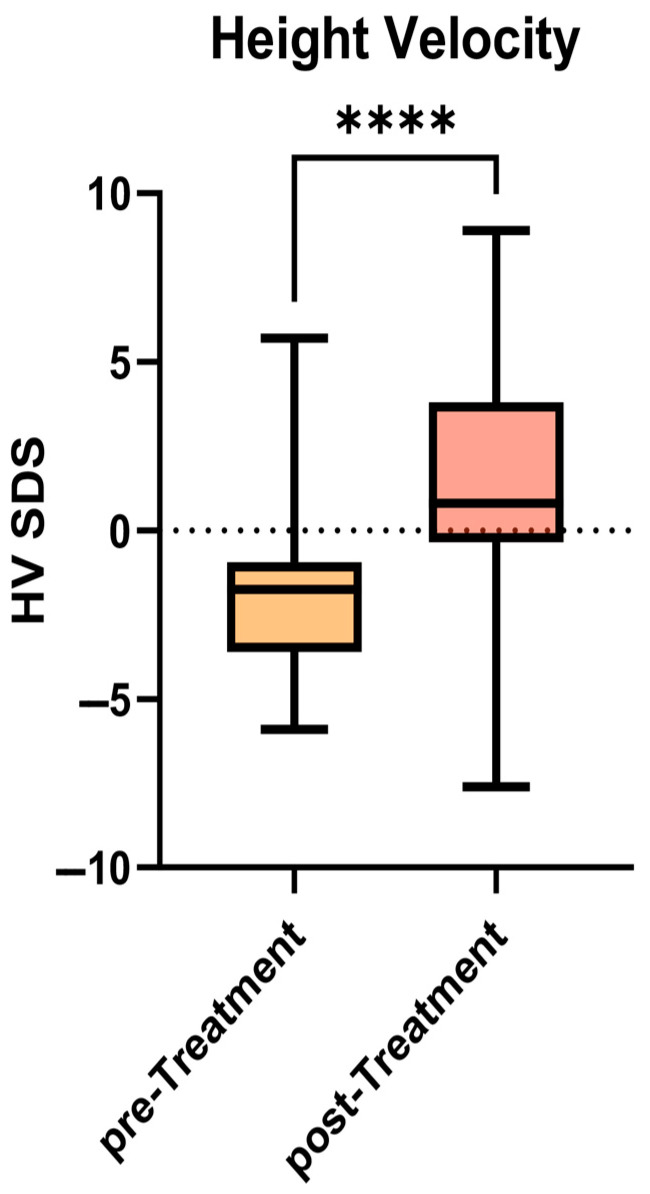
Comparison of HV SDS before and after six months of treatment with ETI. (**** = extremely significant, *p* < 0.0001).

However, as shown in Figure 2, significance was reached when comparing the mean HV calculated between T(−6) and T0 (4.2 ± 2.0 cm/year; −1.96 ± 2.4 SDS) with the HV between T0 and T(+6) (7.1 ± 3.0 cm/year; +1.5 ± 3.7 SDS) (*p* < 0.0001).

**Figure 3:**
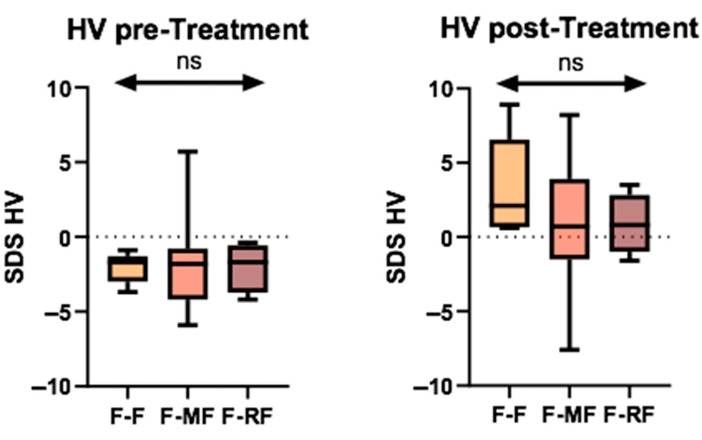
Comparison of HV SDS among the three genetic groups before and after treatment. (ns = not significant).

With regard to genetic characterization, the population was stratified into three different groups: 5 patients (20.8%) were homozygous for the F508del mutation (F/F), 11 (45.8%) had a minimal function mutation combined with the F508del (F/MF), and 8 (33.3%) had a residual function mutation (F/RF). We conducted a comparison of the median HVs among the three groups, both before and after treatment. However, no significant difference was observed in any analyses (Figure 3).

**Figure 4:**
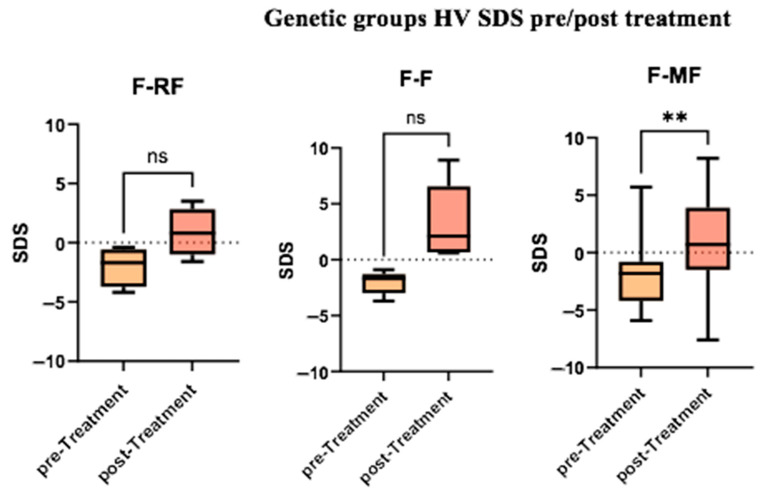
Comparison of median HVs before and after treatment within each genetic group. (ns = not significant, **= very significant, *p* < 0.01).

Additionally, we performed intra-group comparisons, assessing the median HVs before and after treatment within each group. The difference was statistically significant only within the F/MF group (Figure 4).

Lumbar and total body DXA scans were performed on all subjects. The median lumbar spine BMD was 0.570 g/cm^2^ [IQR: 0.520–0.620], with a corresponding mean z-score of −0.07 ± 0.86. None of the patients had reduced bone mineral density or osteoporosis according to the International Society for Clinical Densitometry’s definition. Total body DXA scans confirmed the lumbar data, showing a TBLH mean BMD of 0.650 ± 0.080 g/cm^2^ and a mean z-score of −0.07 ± 0.88. Furthermore, total body DXA scans allowed us to assess body composition. The median fat mass was 25.3% [IQR: 20.8–27.6], while the median lean mass was 72.2% [IQR: 69.9–76.58].

**Figure 5:**
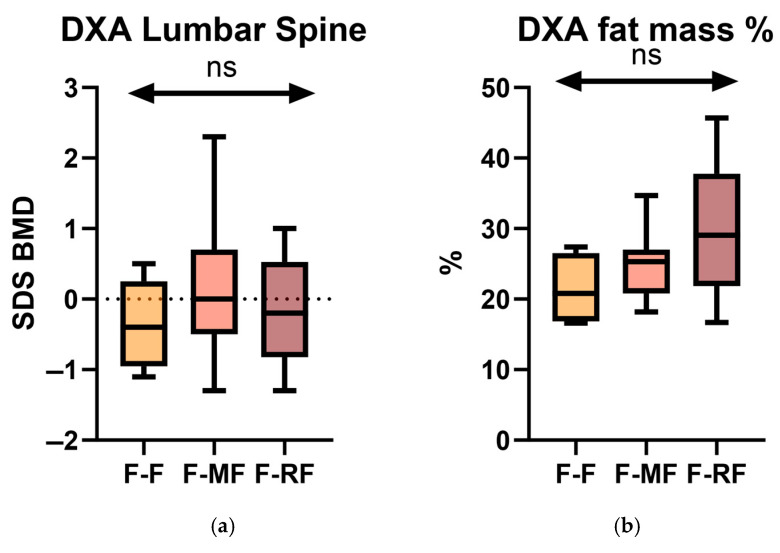
Comparison of lumbar spine BMD SDS (**a**) and fat mass % (**b**) obtained by DXA scans among the 3 genetic groups. (ns = not significant).

By using the stratification into the three genetic groups (F/F, F/MF, and F/RF), we investigated differences in DXA parameters at baseline (T0). Specifically, we conducted a statistical analysis comparing the lumbar spine BMD SDS and fat mass among the three groups (Figure 5).

However, no statistically significant differences were observed at T0.

**Figure 6:**
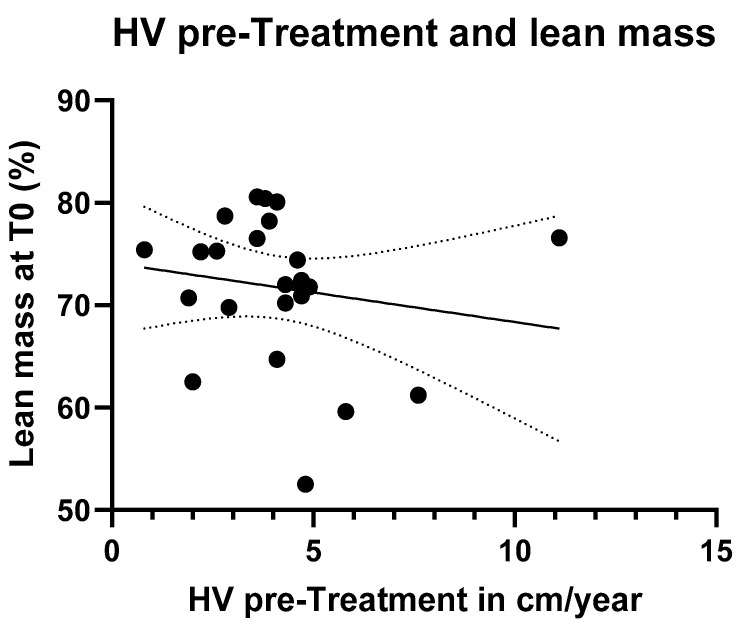
Simple linear regression between HV before treatment and lean mass.

A simple linear regression was performed between HVs (calculated in the six months before treatment) and lean mass (obtained by DXA scans) percentage. Nevertheless, no statistically significant difference was found (Figure 6).

We obtained data on body composition by performing BIA measurements. BIA measurements were conducted on 17 out of the total number of patients, accounting for 70.8%. The median fat mass in this case was 19.2% [IQR: 16.5–23.4].

The median of 1STST at baseline evaluation was 44.6/minute [IQR: 38.5–54.2], with the test conducted on 21 out of 24 patients (87.5%).

## 4. Discussion

To the best of our knowledge, the effect of ETI treatment on HV has not been previously described. This is the first report on a small group of young participants with CF treated with the highly effective modulator in real-life in a single center setting.

However, until now, only the impact of ETI on body weight, BMI, and height-for-age z-score has been analyzed in the literature, as was the case in the 24-week phase 3 trial on patients aged 6–11 years [14]. BMI, BMI z-score for age, weight, weight z-score for age, and height increased over the 24-week treatment period without reaching a plateau, while height z-score for age was maintained [14]. No comparisons have been performed with the six months prior to the start of ETI by trial data.

In our analysis, we observed a rapid increase in HV over six months, while SDS of both BMI and body weight did not change significantly in a statistically meaningful manner.

Interestingly, we also found that this effect was more pronounced in pwCF with minimal function mutations. In fact, as reported by other authors, the effect of CFTR modulator therapies on anthropometric parameters depends on the CFTR mutations and the type of modulation therapy used [15]. Regarding previous modulators, Ivacaftor, as expected, did have an effect on growth in the pwCF with gating mutations [15]. Lumacaftor in combination with Ivacaftor, indicated for pwCF who are homozygous for F508del, appeared to have a positive effect on stature-for-age in patients aged 2–5 years [16].

The combination of Ivacaftor and Tezacaftor did not affect growth in pwCF who were homozygous for the F508del CFTR, despite the effect on lung function [17]. The same results were found in pwCF 12 years and older who were heterozygous for the F508del mutation over an 8 week analysis [18].

Further research is needed to fully understand the effects of ETI on nutritional status in all classes of CFTR mutations. However, based on previous data for other modulators, the improvement in auxological parameters is likely to be related to the effect of CFTR correction at the gastrointestinal level, reduced resting energy expenditure, improved fat absorption due to reduced hydrochloric acid secretion, and reduced intestinal inflammation [15]. Another pathological explanation could be the effect of CFTR protein correction on growth hormone regulation, reduced IGF-1 levels, and peripheral GH resistance [19]. Its effect on the GH axis could also be indirect through the effect of some anti-inflammatory cytokines such as IL-6 on the JAK-STAT axis [20]. Other effects of ETI include the impact on pancreatic insufficiency and chronic insulin deficiency, which also affect the nutritional status [19].

As expected, our results showed a greater HV increase in the F/F and F/MF patient groups, both of which are characterized by more severe CFTR genotypes and universal exocrine pancreatic insufficiency [21]. The strongest effect observed on HV in pwCF with a minimal function mutation could be explained by either the larger group in our study population (15 pts, 62.5%) or the fact that F508del homozygous patients received previous modulator therapy (lumacaftor + ivacaftor), which may have mitigated the effect of ETI. The lower HV increase in the F/RF group is justified by the fact that these patients had milder CF disease and often preserved pancreatic function, which is known to result in an overall better clinical condition at baseline [22]. In fact, pancreatic insufficiency can have an effect already in utero, leading to obstruction of the pancreatic ducts, pancreatic inflammation, and pancreatic fibrosis, with major effects on growth [23].

The new highly effective CFTR modulators, therefore, open up new perspectives in CF, which has traditionally been characterized by malnutrition, poor growth and low weight, and nowadays also by excessive body weight, as reported in the literature [24].

The DXA scans revealed that none of the patients, who were predominantly prepubertal and thus with no effect of sexual hormones on the bone, had reduced bone mineral density. This indicates that pwCF, at least during childhood, commonly have good bone health. To the best of our knowledge, no data were reported in the literature about BMD and body composition in a population of children with CF aged 6 to 11 years.

Concerning body composition, we assumed that both higher lean mass and HV are indicators of well-being. Therefore, we hypothesized a possible positive correlation between these two parameters, meaning that a higher percentage of lean mass would be associated with greater growth velocity. We analyzed the relationship between baseline lean mass (T0) and HV during the pre-treatment phase using simple linear regression. However, no statistically significant difference was observed between the two variables, likely due to the small sample size and the short follow-up period. Another possible reason could be that these variables are influenced by multiple factors, and an additional confounding variable may have unexpectedly influenced the analysis. Nonetheless, it is possible that with a longer follow-up period, this relationship could become more evident.

## 5. Conclusions

In conclusion, our study demonstrates that ETI is a powerful tool, not only for improving respiratory function, particularly in the pediatric population who generally begin with better baseline status, but also for enhancing HV during a critical stage of development. As evidenced in the literature, growth failure in childhood typically precedes pulmonary decline, influencing both reduced adult height and impaired lung development [25]. Specifically, it has been reported that children with a height above the 50th percentile at 6–7 years of age exhibit better FEV1 values, independent of BMI, suggesting that linear growth plays a critical role in lung function and is associated with increased survival rates [26,27,28]. By identifying new growth trajectories through ETI, we can hypothesize that in the pediatric population, there exists a window of opportunity to enhance respiratory function into adulthood [28].

The strength of our study lies in being the first to analyze a novel parameter of well-being that has not yet been explored in real-life settings. The limitations include the small sample size and the absence of the analysis of the GH axis with the assessment of GH secretion and IGF-1 levels, and their effects on both body composition and inflammation. Moreover, we were unable to include comparisons with age-matched children without cystic fibrosis (CF) or with children with CF not receiving ETI, which would have represented valuable comparators. We acknowledge this as a limitation and plan to explore these comparisons in future studies with a broader cohort and extended follow-up.

## Figures and Tables

**Table 1:** Auxological data expressed as SDS (mean ± SD).

	Height in cm (SDS)	Weight in Kg (SDS)	BMI in Kg/m^2^ (SDS)
T(−6)	129.3 ± 10.8 (+0.05 ± 0.89)	27.9 ± 6.6 (−0.27 ± 0.80)	16.5 ± 2.0 (−0.41 ± 0.96)
T0	131.5 ± 10.6 (−0.07 ± 0.94)	29.1 ± 6.7 (−0.35 ± 0.82)	16.6 ± 1.8 (−0.41 ± 0.82)
T(+6)	135.3 ± 10.5 (+0.03 ± 0.96)	31.0 ± 6.8 (−0.35 ± 0.74)	16.8 ± 1.8 (−0.45 ± 0.78)

**Table 2:** Demographic characteristics of patients (24 pt).

**Population**	24
**Female/male**	12/12
**F508del homozygous, n (%)**	5 (20.8)
**F508del/minimal function, n (%)**	11 (45.8)
**F508del/residual function, n (%)**	8 (33.3)
**Age (years, median)**	8.7
**Meconium ileus (%)**	3 (12.5)
**Pancreatic insufficiency (%)**	22 (91.7)
**Sweat chloride concentration (mmol/L, median)**	91.08
**Fev1 > 70% (%)**	24 (100)
**Pseudomonas aeruginosa colonization (%)**	4 (16.7)
**Non tuberculous mycobacteria colonization (%)**	2 (8.3)
***Burkholderia cepacia* colonization (%)**	0 (0)
**Cystic fibrosis liver disease (%)**	10 (41.7)
**Cystic fibrosis related diabetes (%)**	0 (0)
**Severe cystic fibrosis liver disease (%)**	0 (0)
**Average number of exacerbations per year before eti (intravenous antibiotics)**	0.3
**Average number of exacerbations per year before eti** **(oral antibiotics)**	0.3
**Non invasive ventilation (%)**	0 (0)
**Compassionate use of ETI (%)**	0 (0)
**Patient naïve of CFTR modulators**	19 (79.2)

## Data Availability

The original contributions presented in this study are included in the article. Further inquiries can be directed to the corresponding author.

## Abbreviations

The following abbreviations are used in this manuscript:

pwCF	People with cystic fibrosis
CFTR	CF transmembrane conductance regulator
CF	Cystic fibrosis
HV	Height velocity
SDS	Standard deviation scores
GH	Growth hormone
IGF-1	Insulin-like growth factor 1
ETI	Elexacaftor/Tezacaftor/Ivacaftor
BMI	Body mass index
BMD	Bone mineral density
DXA	Dual-energy X-ray absorptiometry
BIA	Bioelectrical impedance analysis
TBLH	Lumbar and Total Body Less Head
1STST	One-minute sit-to-stand test
